# Accuracy Improvement of IOL Power Prediction for Highly Myopic Eyes With an XGBoost Machine Learning-Based Calculator

**DOI:** 10.3389/fmed.2020.592663

**Published:** 2020-12-23

**Authors:** Ling Wei, Yunxiao Song, Wenwen He, Xu Chen, Bo Ma, Yi Lu, Xiangjia Zhu

**Affiliations:** ^1^Department of Ophthalmology and Eye Institute, Eye & ENT Hospital, Fudan University, Shanghai, China; ^2^National Health Commission Key Laboratory of Myopia, Fudan University, Shanghai, China; ^3^Key Laboratory of Myopia, Chinese Academy of Medical Science, Shanghai, China; ^4^Shanghai Key Laboratory of Visual Impairment and Restoration, Shanghai, China; ^5^New York University Shanghai, Shanghai, China; ^6^Shanghai Aier Eye Hospital, Shanghai, China; ^7^Department of Ophthalmology, Ninth People's Hospital of Shanghai Jiaotong University, Shanghai, China

**Keywords:** machine learning, refractive error, myopia, intraocular lens, IOL power calculation

## Abstract

**Purpose:** To develop a machine learning-based calculator to improve the accuracy of IOL power predictions for highly myopic eyes.

**Methods:** Data of 1,450 highly myopic eyes from 1,450 patients who had cataract surgeries at our hospital were used as internal dataset (train and validate). Another 114 highly myopic eyes from other hospitals were used as external test dataset. A new calculator was developed using XGBoost regression model based on features including demographics, biometrics, IOL powers, A constants, and the predicted refractions by Barrett Universal II (BUII) formula. The accuracies were compared between our calculator and BUII formula, and axial length (AL) subgroup analysis (26.0–28.0, 28.0–30.0, or ≥30.0 mm) was further conducted.

**Results:** The median absolute errors (MedAEs) and median squared errors (MedSEs) were lower with the XGBoost calculator (internal: 0.25 D and 0.06 D^2^; external: 0.29 D and 0.09 D^2^) vs. the BUII formula (all *P* ≤ 0.001). The mean absolute errors and were 0.33 ± 0.28 vs. 0.45 ± 0.31 (internal), and 0.35 ± 0.24 vs. 0.43 ± 0.29 D (external). The mean squared errors were 0.19 ± 0.32 vs. 0.30 ± 0.36 (internal), and 0.18 ± 0.21 vs. 0.27 ± 0.29 D^2^ (external). The percentages of eyes within ±0.25 D of the prediction errors were significantly greater with the XGBoost calculator (internal: 49.66 vs. 29.66%; external: 78.28 vs. 60.34%; both *P* < 0.05). The same trend was in MedAEs and MedSEs in all subgroups (internal) and in AL ≥30.0 mm subgroup (external) (all *P* < 0.001).

**Conclusions:** The new XGBoost calculator showed promising accuracy for highly or extremely myopic eyes.

## Introduction

High myopia has become a worldwide epidemic, with a predicted prevalence of 10% of the world population by the year 2050 (1). It often leads to significant visual impairment or even blindness (2). Patients with high myopia also have a higher risk of developing cataracts and undergo cataract surgeries at an earlier age (3–5). Therefore, the accurate IOL power calculation for these eyes is an important issue.

However, highly myopic eyes often experience hyperopic refractive surprise after cataract surgery (6, 7), despite the use of partial coherence interferometry, which could eliminate biometric errors (8, 9). Therefore, choosing appropriate formulas to reduce refractive errors becomes crucial in these eyes.

Fourth-generation formulas, such as the Barrett Universal II (BUII) (10), Olsen, and Hill-Radial Basis Function (RBF) formulas, offered promising outcomes for highly myopic eyes (11, 12). In particular, the BUII formula may show the greatest accuracy for myopic eyes (13–15). For eyes with ALs > 24.5 mm, the BUII formula presented the highest percentage of eyes within ±0.50 D of the prediction error (PE) (82.1% on average) (16). However, the accuracy of the BUII formula decreased sharply when we included more eyes with extreme myopia (AL > 28.0 mm), as the percentage of eyes within ±0.50 D of the PE decreased to 70%, and the percentage within ±0.25 D of the PE was only 25% (17). Therefore, accurate prediction of IOL power for eyes with high or extreme myopia remains challenging.

The purpose of this study was to develop a new calculator using the XGBoost machine learning regression technique that incorporates several clinical features, including the BUII formula results, to improve the accuracy of IOL power prediction for highly or extremely myopic eyes.

## Methods

### Patients

The Institutional Review Board of the Eye and Ear, Nose, and Throat (ENT) Hospital of Fudan University (Shanghai, China) approved this study. The study adhered to the tenets of the Declaration of Helsinki and was registered at www.clinicaltrials.gov (accession number NCT02182921). Signed informed consents for the use of their clinical data were obtained from all participants before cataract surgery.

Data of 1,450 highly myopic eyes from 1,450 patients who had uneventful cataract surgery at our hospital were collected to develop and validate the models (internal dataset). Data from the Shanghai Aier Eye Hospital and the Ninth People's Hospital of Shanghai Jiaotong University were collected as an external test dataset, including another 114 highly myopic eyes from 114 patients.

The inclusion criteria were: (1) axial length (AL) >26.0 mm; (2) preoperative biometry obtained using IOLMaster 700 (version 1.80) or IOLMaster 500 (version 7.7, Carl Zeiss Meditec AG, Jena, Germany); (3) uneventful cataract surgery with credible postoperative (≥1 month) manifest refraction outcomes; and (4) best corrected distance visual acuity (BCVA) taken by a Snellen chart at 2.5 m more than 1 month after surgery. The exclusion criteria were: (1) severe corneal opacity; (2) severe maculopathy, which was defined the fundus photograph (Optos-200Tx Ultra-Widefield Retinal Imaging System, Optos, Dunfermline, United Kingdom) results reaching category 4 according to the international photographic grading system for myopic maculopathy proposed by Ohno-Matsui et al. (18), or the OCT exam (Spectralis OCT; Heidelberg Engineering, Heidelberg, Germany) revealed severe lesions such as the macular hole, choroidal neovascularization, atrophy, etc.; and (4) eyes with ocular trauma or other diseases that may influence the accuracy of manifest refraction.

The IOL models in the internal dataset included MCX 11 ASP, Rayner, 409MP, HOYA, ZCB00, SN60WF, and ZMB00, while the external dataset included MCX 11 ASP, SN60WF, and ErgomaX.

The A constants were obtained from the User Group for Laser Interference Biometry website (ocusoft.de/ulib/index.htm) for SRK/T formula, after which the constants were input into the website of BUII formula and lens factors were automatically generated.

### Dataset Preparation

The project included three main parts: dataset preparation, model design, and training and evaluation.

Data from our hospital were set as the internal dataset, while 20% of the eyes were split randomly into a test dataset, and the remainders were used as the train and validation datasets. Data from the other hospitals were set as an external test dataset.

Actual postoperative refraction measured more than 1 month after cataract surgery was set as the training target, with others listed below set as features of machine learning. The four types of features were: (1) demographic information, which was the patient age; (2) biometric data, which included AL, corneal curvature (flattest and steepest K value, namely K1 and K2), steepest and flattest meridian, and anterior chamber depth (ACD, measured from epithelium to lens). We also included two additional parameters: $\frac{1}{AL}\;$ and $\frac{1}{\left(K1+K2\right)}$, by feature transformation during preprocessing; (3) the power of the implanted IOL model and its A constant; and (4) the predicted refraction of the implanted IOL back-calculated using the BUII formula.

### Modeling

XGBoost is an algorithm in which new models are created that predict the residuals of prior models and are then added together to make the final prediction (19). Using an internal dataset, the XGBoost model was compared with another two regression models, including Random Forests (RF) (20), and linear support vector machine (SVM) regressor (21), and the one with best prediction outcomes was adopted for further analysis.

The clinical features in the training dataset were input into the three machine learning models to predict the postoperative refractions. Random search with 3-fold cross-validation was used to determine the hyperparameters of the models, which were randomly selected within a range. Through 500 repetitions, the optimal hyperparameters with the highest validation scores were chosen for our model. The hyperparameters used in our study for XGBoost were learning_rate (0,0.1], n_estimator (300, 700), max_depth (2,3), gamma (1), subsample [0.7, 0.9], and colsample_bytree [0.7, 0.9]; for RF were n_estimators [1,200], min_samples_leaf [1,1000], and max_features (1, 12); for linear SVM were epsilon [0,1] and C [0,10].

### Evaluation

To evaluate the precision of the model, the trained and tuned prediction model was used to predict postoperative refraction using the internal and external test datasets. The PE was defined as the actual postoperative refraction (spherical equivalent) minus the predicted postoperative refraction (back-calculated with the actually implanted IOL power). The median absolute errors (MedAEs) (22), median squared errors (MedSEs), mean absolute errors (MAEs), mean squared errors (MSEs), and the percentages of eyes within ±0.25, ±0.50, ±0.75, and ±1.00 D of the PE were calculated and compared between the new calculator and the BUII formula. Furthermore, a comparison between the new calculator and the RBF 2.0 formula was further conducted with part of the data from the internal and external test datasets, while only cases with −2.5 to 1 D refractive targets or AL <35 mm can be calculated with the RBF 2.0 calculator according to its user guide (23).

**Figure 1:**
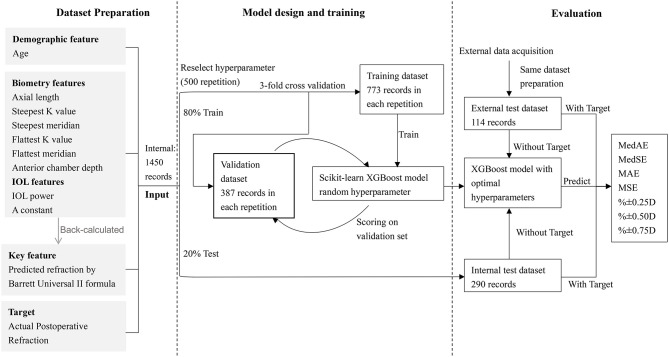
The flow diagram of the XGBoost model.

The flow diagram of our model was demonstrated in Figure 1. To validate the stability and generalizability of the model, the training dataset and internal test dataset were randomly split with a fixed proportion (80:20%) with 100 repetitions. In each repetition, a new model was established based on the training dataset and was evaluated using the same metrics and a separate test dataset.

We also conducted subgroup analysis stratified by ALs (26.0–28.0, 28.0–30.0, and ≥30.0 mm) with both test datasets. Eyes with AL ≥28.0 mm were defined as extremely myopic eyes. The accuracies of our calculator and the BUII formula were compared in each subgroup using the evaluation metrics described above.

### Application

A free website for our XGBoost calculator was developed for online calculations (*zhuformula.com*, with user guide provided).

### Statistical Analyses

Statistical analyses were performed with SPSS software (version 11.0, SPSS, Inc.). Continuous variables were described as the mean ± standard deviation. The student's *t*-test was used to compare the continuous variables and the χ^2^ test was used to compare categorical variables. Outcome measurements with abnormal distributions were compared with the Wilcoxon signed-rank test (two groups) or the Kruskal-Wallis test (more than two groups). Linear-by-linear associations (two groups) and Kaplan-Merier test (more than two groups) were used to compare the distributions of the PE. A *P*-value of <0.05 was considered statistically significant.

## Results

### Demographics and Ocular Characteristics

The demographic data for the eyes in the internal and external datasets are shown in Table 1. There were no statistically significant between-group differences in age, sex, and laterality. However, the external dataset had longer AL, larger K1, and K2, and lower implanted IOL power. Extremely myopic eyes comprised 62.07% (180/290) of all eyes in the internal test dataset and 79.82% (91/114) in the external test dataset.

**Table 1:** Demographics of the internal and external datasets.

**Parameters**	**Internal dataset (*n* = 1,450)**	**External dataset (*n* = 140)**	***P*-value**
**Age (y)**			
Mean ± SD	61.32 ± 9.25	62.61 ± 7.90	>0.05
Range	25–87	44–85	
Female No. (%)	794 (54.8%)	80 (57.1%)	>0.05
Eye (OD/OS)	760/690	62/52	>0.05
**Axial length (mm)**			
Mean ± SD	29.36 ± 2.18	29.87 ± 2.13	0.043
Range	26.01–36.46	26.05–35.99	
**K1 (D)**			
Mean ± SD	43.00 ± 1.97	43.64 ± 1.50	0.002
Range	32.02–48.38	39.38–46.62	
**K2 (D)**			
Mean ± SD	44.16 ± 2.06	44.80 ± 1.55	0.002
Range	32.96–50.15	41.31–48.64	
**IOL power (D)**			
Mean ± SD	9.56 ± 5.61	7.12 ± 4.70	<0.001
Range	−8.0 to 27.5	−4.0 to 17.0	

*SD, standard deviation; D, diopter*.

*Student's t-test was used to compare the continuous variables and the χ^2^ test was used to compare categorical variables between the internal and external datasets*.

### Comparisons of Accuracy

**Figure 2:**
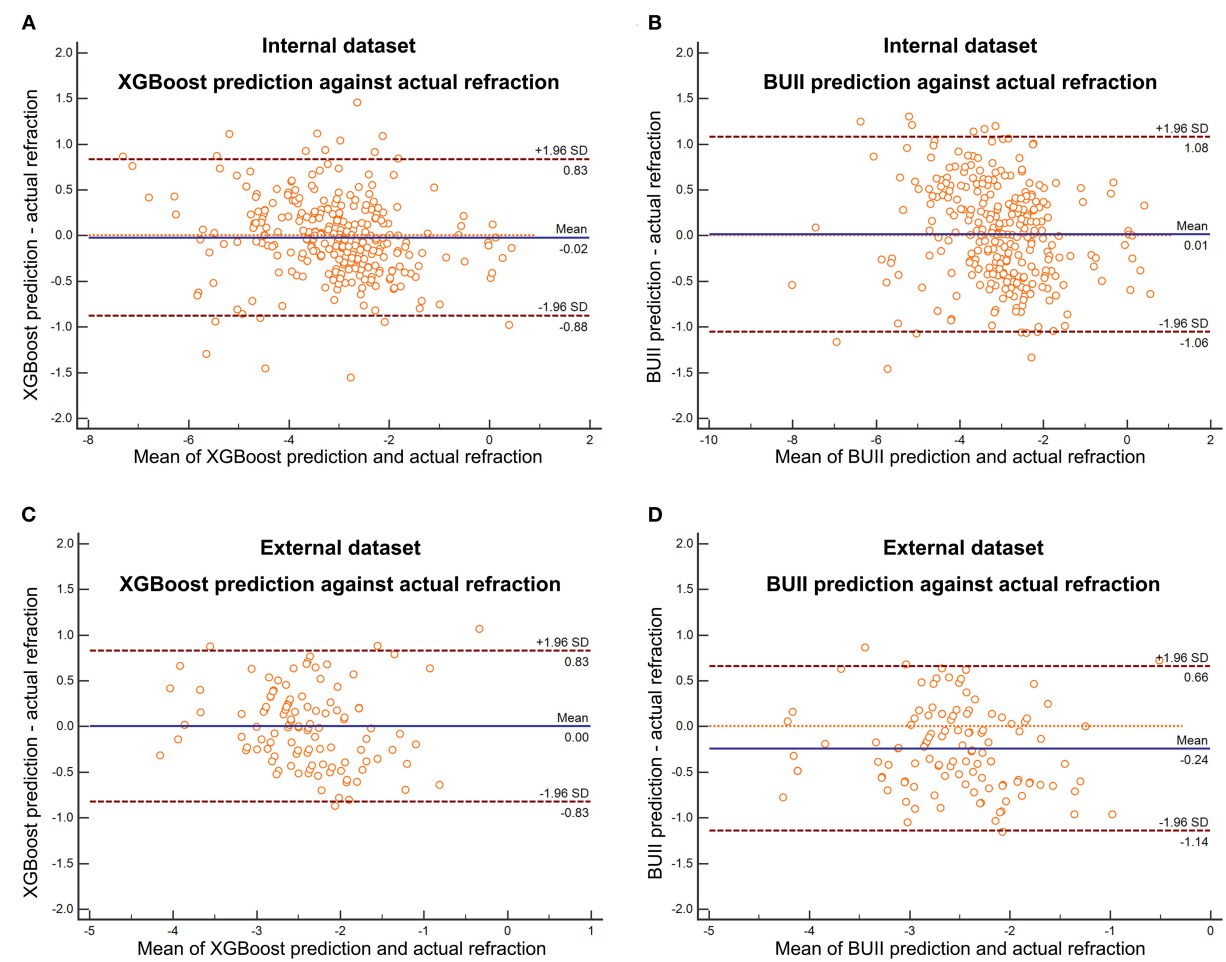
The Bland-Altman plots with actual postoperative refraction against the XGBoost prediction in the internal **(A)** and external **(C)** test dataset or Barrett Universal II formula (BUII) prediction in the internal **(B)** and external **(D)** test dataset.

Comparisons of three regression models demonstrated that the XGBoost outperformed RF and linear SVM (*P* < 0.01, Table 2). We then developed our XGBoost calculator and compared its prediction results with the BUII formula in both test datasets. The Bland-Altman plots with actual postoperative refraction against the XGBoost or the BUII outputs were demonstrated in Figure 2, and most points were within the agreement limits. The summary of accuracy outcomes was demonstrated in Table 3. The mean predicted refractions in the internal test dataset were −3.09 D (range −6.88 to 0.37 D) by the XGBoost calculator, and −3.06 D (range −8.29 to 0.58 D) by the BUII; while in the external test dataset were −2.41 D (range −4.32 to 0.20 D) by the XGBoost calculator, and −2.66 D (range −4.65 to −0.15 D) by the BUII. The MedAEs and MedSEs were significantly lower with the XGBoost calculator in two test datasets (internal: MedAE: 0.25 vs. 0.42 D and MedSE: 0.06 vs. 0.17 D; external: MedAE: 0.29 vs. 0.42 D and MedSE: 0.09 vs. 0.18 D; all *P* ≤ 0.001). The MAEs with our XGBoost calculator vs. the BUII were 0.33 ± 0.28 vs. 0.45 ± 0.31 D in internal test dataset, and 0.35 ± 0.24 vs. 0.43 ± 0.29 D in external test dataset. The MSEs were 0.19 ± 0.32 vs. 0.30 ± 0.36 D^2^ in internal test dataset, and 0.18 ± 0.21 vs. 0.27 ± 0.29 D^2^ in external test dataset. As for the 100 times random cross-validation analyses, the MAEs of our XGBoost formula were consistently lower in all rounds of modeling and calculation (Supplementary Figure 1).

**Table 2:** Comparison regression models including the XGBoost, Linear Support Vector Machine, and Random Forest using the internal test dataset.

**Parameters**	**XGBoost**	**Random Forest**	**Linear SVM**	***P*-value**
MedAE (D)	0.25	0.30	0.70	<0.01
MedSE (D^2^)	0.06	0.09	0.49	<0.01
MAE (D) ± SD	0.33 ± 0.28	0.38 ± 0.33	0.96 ± 0.90	
MSE (D^2^) ± SD	0.19 ± 0.32	0.25 ± 0.50	1.74 ± 3.14	
Eyes within PE (%)				<0.01
±0.25 D	49.66%	45.17%	20.34%	
±0.50 D	78.28%	75.17%	38.97%	
±0.75 D	90.96%	88.97%	52.76%	
±1.00 D	97.24%	94.14%	63.97%	

*SVM, support vector machine; MedAE, median absolute error; MedSE, median squared error; MAE, mean absolute error; SD, standard deviation; MSE, mean squared error; D, diopter; PE, prediction error*.

*Kruskal-Wallis tests were used to compare the MedAE and MedSE results. Kaplan-Merier test was used to compare the percentages of eyes within ±0.25, ±0.50, ±0.75, and ±1.00 D of PE*.

**Table 3:** Summary of outcomes for the XGBoost calculator and the Barrett Universal II formula for the internal and external test datasets.

**Parameters**	**Internal test dataset (*****n*** **=** **290)**			**External test dataset (*****n*** **=** **114)**		
	**XGBoost**	**BUII formula**	***P*-value**	**XGBoost**	**BUII formula**	***P*-value**
Mean predicted refraction [range]	−3.09 [−6.88, 0.37]	−3.06 [−8.29, 0.58]		−2.41 [−4.32, 0.20]	−2.66 [−4.65, −0.15, ]	
MedAE (D)	0.25	0.42	<0.001	0.29	0.42	0.001
MedSE (D^2^)	0.06	0.17	<0.001	0.09	0.18	0.001
MAE (D) ± SD	0.33 ± 0.28	0.45 ± 0.31		0.35 ± 0.24	0.43 ± 0.29	
MSE (D^2^) ± SD	0.19 ± 0.32	0.30 ± 0.36		0.18 ± 0.21	0.27 ± 0.29	
Eyes within PE (%)			<0.001			0.031
±0.25 D	49.66%	29.66%		43.86%	35.96%	
±0.50 D	78.28%	60.34%		72.81%	58.77%	
±0.75 D	90.69%	84.14%		92.98%	85.09%	
±0.10 D	97.24%	93.45%		99.12%	97.37%	

*BUII, Barrett Universal II; MAE, mean absolute error; MedAE, median absolute error; SD, standard deviation; MSE, mean squared error; MedSE, median squared error; D, diopter*.

*Wilcoxon signed-rank tests were used to compare the MedAE and MedSE results, and linear-by-linear associations were used to compare the distributions of the refractive errors*.

**Figure 3:**
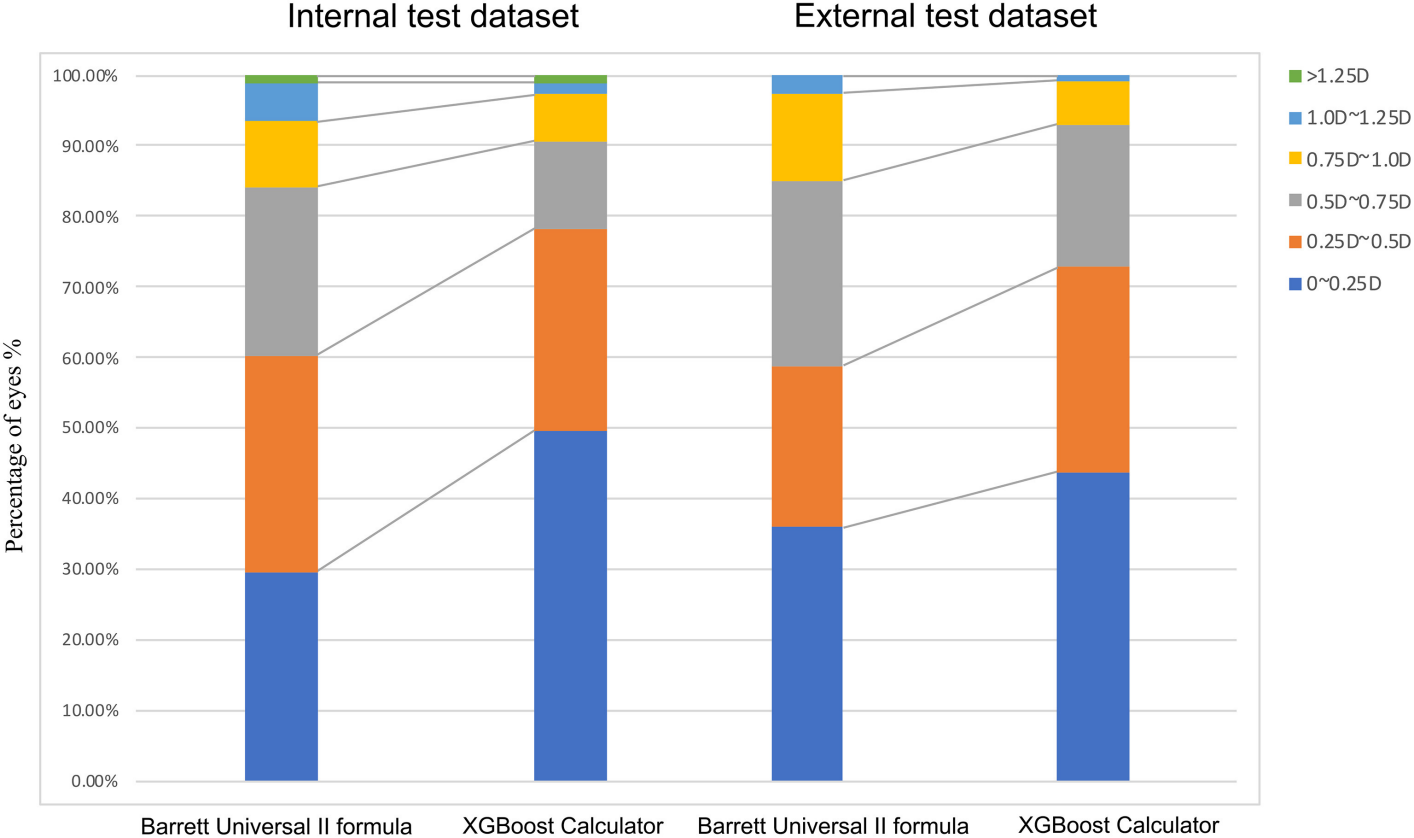
Distribution of eyes within ±0.25 D, ±0.50 D, ±0.75 D, ±1.00 D, or ±1.25 D of the prediction errors in the internal test dataset.

The percentages of eyes within ±0.25, ±0.50, ±0.75, and ±1.00 D of the PE were greater with the XGBoost calculator in the internal (±0.25 D: 49.66 vs. 29.66%; ±0.50 D: 78.28 vs. 60.34%; ±0.75 D: 90.69 vs. 84.14%; ±1.00 D: 97.24 vs. 93.45%; *P* <0.001) and external dataset (±0.25 D: 43.86 vs. 35.96%; ±0.50 D: 72.81 vs. 58.77%; ±0.75 D: 92.98 vs. 85.09%; ±1.00 D: 99.12 vs. 97.37%; both *P* < 0.05) (Table 3 and Figure 3). Furthermore, the new XGBoost calculator also outperformed the RBF 2.0 calculator in the internal and external test datasets (Supplementary Table 1).

### Subgroup Analysis

Table 4 shows the results of the AL subgroup analysis. The MedAEs and MedSEs were significantly lower with the XGBoost calculator than the BUII formula in all subgroups in the internal dataset (all *P* < 0.001) and in the AL ≥30.0 mm subgroup in the external dataset (*P* < 0.001). The mean absolute errors (MAEs) with our XGBoost calculator vs. the BUII in three AL subgroups in internal test dataset were 0.29 ± 0.24, 0.35 ± 0.30, and 0.36 ± 0.30 D, respectively, vs. 0.42 ± 0.29, 0.48 ± 0.35, and 0.46 ± 0.29 D, respectively; while in the AL subgroups of the external test dataset were 0.35 ± 0.26, 0.35 ± 0.24, and 0.34 ± 0.24 D, respectively, vs. 0.35 ± 0.24, 0.38 ± 0.27, and 0.51 ± 0.30 D, respectively. The mean squared errors (MSEs) in the AL subgroups of the internal test dataset were 0.14 ± 0.21, 0.21 ± 0.40, and 0.22 ± 0.35 D^2^, respectively, vs. 0.26 ± 0.32, 0.35 ± 0.45, and 0.30 ± 0.33 D^2^, respectively; while in the AL subgroups of the external test dataset were 0.18 ± 0.25, 0.18 ± 0.21, and 0.17 ± 0.20 D^2^, respectively, vs. 0.18 ± 0.22, 0.21 ± 0.22, and 0.35 ± 0.33 D^2^, respectively.

**Table 4:** Summary of outcomes from axial length subgroup analyses comparing the XGBoost calculator and the Barrett Universal II formula in the internal and external test datasets.

**Subgroups/Parameters**	**Internal test dataset (*****n*** **=** **290)**			**External test dataset (*****n*** **=** **114)**		
	**XGBoost**	**BUII formula**	***P*-value**	**XGBoost**	**BUII formula**	***P*-value**
**AXIAL LENGTH**						
**26.0–28.0 mm**						
Number of eyes	110			23		
MedAE (D)	0.23	0.40	<0.001	0.30	0.38	>0.05
MedSE (D^2^)	0.05	0.16	<0.001	0.09	0.14	>0.05
MAE (D) ± SD	0.29 ± 0.24	0.42 ± 0.29		0.35 ± 0.26	0.35 ± 0.24	
MSE (D^2^) ± SD	0.14 ± 0.21	0.26 ± 0.32		0.18 ± 0.25	0.18 ± 0.22	
**28.0–30.0 mm**						
Number of eyes	82			37		
MedAE (D)	0.25	0.41	<0.001	0.26	0.42	>0.05
MedSE (D^2^)	0.06	0.17	<0.001	0.07	0.18	>0.05
MAE (D) ± SD	0.35 ± 0.30	0.48 ± 0.35		0.35 ± 0.24	0.38 ± 0.27	
MSE (D^2^) ± SD	0.21 ± 0.40	0.35 ± 0.45		0.18 ± 0.21	0.21 ± 0.22	
≥**30.0 mm**						
Number of eyes	98			54		
MedAE (D)	0.35	0.44	<0.001	0.32	0.52	<0.001
MedSE (D^2^)	0.12	0.20	<0.001	0.11	0.27	<0.001
MAE (D) ± SD	0.36 ± 0.30	0.46 ± 0.29		0.34 ± 0.24	0.51 ± 0.30	
MSE (D^2^) ± SD	0.22 ± 0.35	0.30 ± 0.33		0.17 ± 0.20	0.35 ± 0.33	

*XGBoost, new XGBoost calculator; BUII, Barrett Universal II; MedAE, median absolute error; MedSE, median squared error; MAE, mean absolute error; SD, standard deviation; MSE, mean squared error; D, diopter; PE, prediction error*.

*Wilcoxon signed-rank tests were used to compare the MedAE and MedSE results*.

## Discussion

Accurately predicting the IOL power for highly myopic eyes is quite difficult, especially for extremely myopic eyes. Although the BUII formula appears to be the most accurate formula currently available for highly myopic eyes (8, 12, 24), its accuracy decreases when it is applied to extremely myopic eyes (17). Moreover, despite the promising outcomes of the BUII formula in terms of the percentage of eyes within ±0.50 D of the PE (11, 12, 14, 24), the percentage of eyes within ±0.25 D of the PE remains unsatisfied (25.0–38.3%) (14, 15, 17). Therefore, there is still room for improving the BUII formula for extremely myopic eyes. In this study, we developed a new XGBoost IOL calculator that showed improved accuracy in highly myopic eyes. It performed well in a dataset in which more than two-thirds of eyes were extremely myopic, and it greatly improved the percentage of eyes within ±0.25 D of the PE relative to the BUII formula using the internal (49.66 vs. 29.66%) and external (43.86 vs. 35.96%) test datasets.

In long eyes, the PE is generally attributable to two factors, the ocular biometry and the calculation formula itself. In terms of biometry, longer AL, greater ACD, the occurrence of staphyloma (25, 26), and poor fixation stability can influence the measurement of highly myopic eyes (27). However, the use of noncontact partial coherence interferometry can eliminate measurement errors, thus errors from the selected formula itself are of more concern (11, 28). Optimizing the constants of the existing formulas is one solution (29–31), while improving formulas that consider more characteristic variables for highly myopic eyes might be another.

In recent years, several new formulas that incorporate additional factors into the calculation have been developed, and efforts have been made to determine the most accurate formulas in eyes with ALs >26.0 mm (8, 11, 12, 14, 15, 17). The BUII formula seemed to show the best performance, and when used, the percentage of eyes within ±0.50 D of the PE varies from 62.7 to 89.5% (11, 12, 14, 15, 17). However, its refractive errors inevitably increase as AL increases (6). When we included more eyes with extreme myopia in our previous study, only 70% of eyes were within ±0.50 D of the PE using the BUII formula, with a corresponding value of 60.34% in the current study (17). Apart from that, the percentage of eyes within ±0.25 D of BUII formula was still unsatisfied in highly myopic eyes [25.0% (4), 34.7% (15), or 38.3% (14)]. Therefore, the BUII formula can still be improved for highly or especially extremely myopic eyes. On these grounds, we used machine learning to develop a new calculator to improve the BUII formula for long eyes.

Machine learning and data-driven approaches are becoming more important in ophthalmology (32). The RBF formula is another widely-used formula based on artificial intelligence. Although 86.6% of highly myopic eyes were within ±0.50 D of the PE using the latest version (24), their average AL of the included eyes was 27.72 mm (maximum: 32.36 mm) (24), indicating the validation of the RBF formula in extremely long eyes was still inadequate. In our study, the average AL was >29.00 mm (maximum: 36.46 mm), and more promising accuracy of the XGBoost calculator was also found over the RBF 2.0 formula. Therefore, our XGBoost calculator may be more reliable for extremely long eyes. Moreover, the RBF 2.0 formula is only used in cases where the postoperative target outcome is within −2.5 to 1 D (23). Its implementation is refined when < -2.5 D myopic refractive targets were scheduled for extremely long eyes. Compared with the RBF formula, our calculator might be more useful for IOL power prediction in highly or extremely myopic eyes.

In the present study, it is noteworthy that similar outcomes were also found using the external test dataset, which contained a greater proportion of extremely myopic eyes (79.82%), indicating the generalizability potential of our calculator. Furthermore, when the training and internal test datasets were subjected to 100 rounds of random splitting, our calculator showed high stability with more promising accuracy than the BUII formula. Using our website, surgeons need only enter the IOL power and the predicted refractions calculated with the BUII formula, together with the other clinical feature data required, to yield more accurate predictions of IOL power, and thereby assist surgeons with surgical planning for highly or extremely myopic eyes. Moreover, using the same algorithm, we are willing to further include more data of low or moderate myopic eyes to train the model, and thus further expand the scope of application for our calculator in the future.

In conclusion, we developed a new XGBoost machine learning-based calculator, which demonstrated good accuracy for IOL power prediction in highly and extremely myopic eyes.

## Data Availability Statement

The raw data supporting the conclusions of this article will be made available by the authors, without undue reservation.

## Ethics Statement

The studies involving human participants were reviewed and approved by the Institutional Review Board of the Eye and Ear, Nose, and Throat (ENT) Hospital of Fudan University (Shanghai, China). The patients/participants provided their written informed consent to participate in this study.

## Author Contributions

LW: collected data, performed analyses, and wrote the manuscript. YS: programmed model and performed analyses. WH: collected data and performed analyses. XC and BM: collected data. YL: gained the fund and supervised the process. XZ: revised the manuscript, gained the fund, and supervised the process. All authors contributed to the article and approved the submitted version.

## Conflict of Interest

The authors declare that the research was conducted in the absence of any commercial or financial relationships that could be construed as a potential conflict of interest.
