# Supplementary material for: Accuracy Improvement of IOL Power Prediction for Highly Myopic Eyes With an XGBoost Machine Learning-Based Calculator
Source: Front Med (Lausanne). 2020 Dec 23;7:592663. doi: 10.3389/fmed.2020.592663 (PMC7793738; doi:10.3389/fmed.2020.592663)
Supplement: Supplementary file 1 [file Table_1.pdf]

Supplemental Table 1. Summary of outcomes for the XGBoost calculator and the RBF 2.0 formula.

Parameters	Internal test dataset (n=205)			External test dataset (n=99)		
	**XGBoost**	**RBF 2.0**	***P* value**	**XGBoost**	**RBF 2.0**	***P* value**
MedAE (D)	0.25	0.27	<0.001	0.33	0.34	<0.001
MedSE (D^2)	0.06	0.07	<0.001	0.11	0.12	<0.001
MAE (D) ± SD	0.30 ± 0.24	0.38 ± 0.51		0.37 ± 0.24	0.39 ± 0.28	
MSE (D^2) ± SD	0.14 ± 0.25	0.40 ± 2.85		0.20 ± 0.22	0.23 ± 0.31	
Eyes within PE (%)			<0.001			<0.001
± 0.25 D	51.22%	47.80%		39.39%	37.37%	
± 0.50 D	82.93%	77.56%		68.69%	70.71%	
± 0.75 D	95.12%	89.76%		91.92%	87.88%	
± 0.10 D	98.54%	96.59%		98.99%	96.97%	

RBF, Hill-Radial Basis Function formula; MAE, mean absolute error; MedAE, median absolute error; SD, standard deviation; MSE, mean squared error; MedSE, median squared error; D, diopter.

Wilcoxon signed-rank tests were used to compare the MedAE and MedSE results, and linear-by-linear associations were used to compare the distributions of the refractive errors.
