# Supplementary material for: Accuracy Improvement of IOL Power Prediction for Highly Myopic Eyes With an XGBoost Machine Learning-Based Calculator
Source: Front Med (Lausanne). 2020 Dec 23;7:592663. doi: 10.3389/fmed.2020.592663 (PMC7793738; doi:10.3389/fmed.2020.592663)

Supplemental Figure 1. Mean absolute errors in 100 rounds of random splitting of the training and internal test datasets.

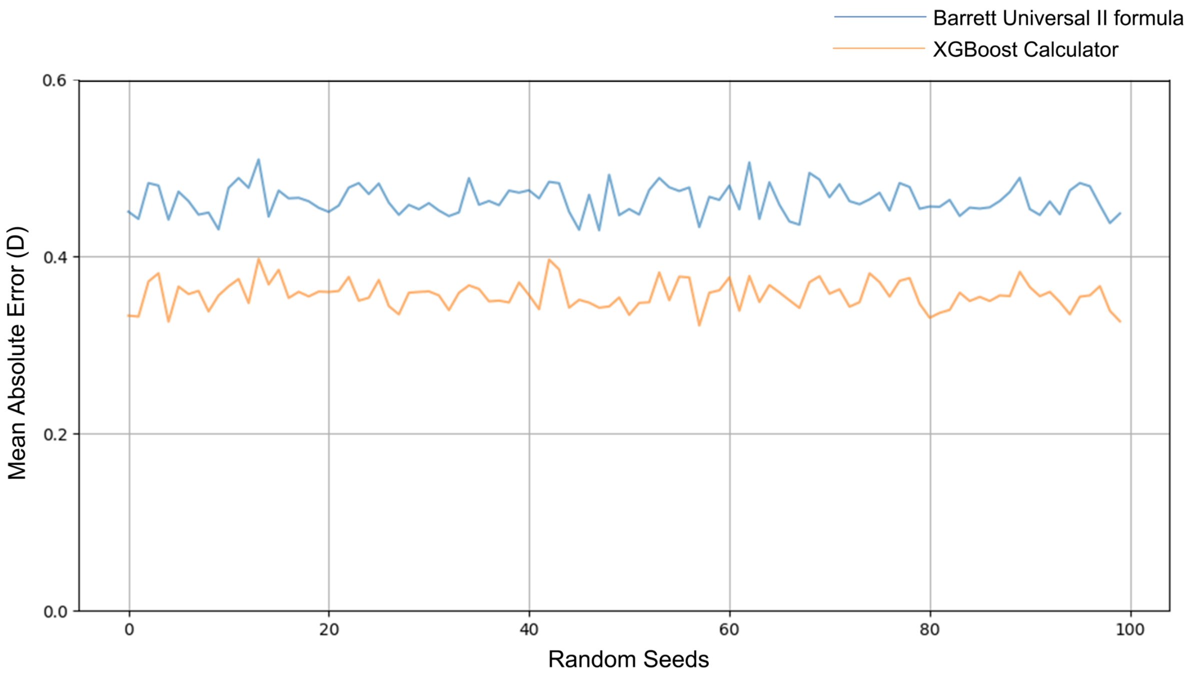

Supplement: Supplementary file 2 [file Image_1.pdf]
